# The Beating Heart of Untapped Business Opportunities for Additive Manufacturing

**DOI:** 10.12688/openreseurope.16270.1

**Published:** 2023-09-07

**Authors:** Isabel Froes, David Struthers, Ciro Malacarne, Matteo Perini, Maurizio Rossi, Paolo Gregori

**Affiliations:** 1 Copenhagen Business School, Management Society and Communication, Copenhagen Business School, Frederiksberg, 1820, Denmark; 2 Trentino Sviluppo S.p.A., Rovereto, 38068, Italy

**Keywords:** Additive Manufacturing, interdisciplinary, user-driven innovation, makerspaces, healthcare, business

## Abstract

This article presents a case that joins user-driven innovation and additive manufacturing (AM) towards latent business opportunities in the preparation for life threatening operations. Surgeons, confronted with a patient with a delicate heart condition, collaborated with a prototyping facility to print a realistic 3D model of the patient’s aortic aneurysm. The model allowed the surgeons to first study and then experiment to determine the most effective operation procedure before the actual operation, which shortened the surgery time by approximately 70%.

Reducing surgery time creates two forms of value: improving patient outcomes and reducing costs. Shorter times under anesthetic and on cardiopulmonary bypass correlate with better surgical results. Reducing healthcare costs brings broad societal benefits in both publicly and privately funded healthcare systems. We outline a case for makerspaces to capture value by joining their expertise and manufacturing equipment with the needs of nearby healthcare systems for novel business developments.

## Plain language summary

This article discusses how people can use a type of technology called Additive Manufacturing (AM) better known as 3D printing to create new business opportunities. The example explored saw surgeons, who had a patient with a serious heart condition, teaming up with a facility to make a lifelike 3D model of the patient's aortic aneurysm. This model allowed the surgeons to study and then try different procedures before operating on the patient. As a result, the surgery time was reduced by about 70%. Reducing the time it takes for a surgery has two important benefits: it improves the outcome for the patient and it lowers costs. When surgeries are shorter, patients have better results and recover faster. It also helps to lower the overall costs of healthcare, which is good for society as a whole, whether the healthcare system is publicly or privately funded. In this article, we suggest that places called makerspaces can play a role in creating new businesses by using their knowledge and equipment to meet the needs of healthcare systems in their area.

## Introduction

The number of maker communities around the world increased significantly over the last decade, though spaces and places for collaboration and creation have long flourished in both physical and virtual forms ( Ciolfi *et al.*, 2008). What sets apart this latest iteration is the joining of computers and digital fabrication technologies, often with facilitating educational and infrastructure resources ( Shanshan, 2016). They range from local groups to globally connected online environments and are targeted at a wide group of interests, expertise, and ages.

Additive manufacturing (AM), which is part of the mesh of digital fabrication, gained considerable market through affordable 3D printers installed in makerspaces, fablabs, shared studios, and private residences. AM technology grew in popularity as its quality improved and affordability increased. Current AM technology offers production qualities ranging from cheap polylactide (PLA) filaments to expensive titanium, aluminum, and stainless steel suitable for load bearing applications ( Bibb *et al.*, 2015). The increased prevalence of AM came with lofty promises, but it has yet to reach its full potential across value chains by restructuring design processes, production locations, supply chains, and shipping to drive innovation and profitability, while reducing environmental impact ( D’Aveni, 2015).

This article presents a case study of user-driven innovation by a makerspace utilizing AM that originated in the EU Innovation Action iProduce. The case is a collaboration between surgeons and engineers at a makerspace in northern Italy that resulted in an accurate 3D printed model of a patient’s aortic aneurism that surgeons used for preoperative planning to reduce the length of the patient’s surgery. Reducing surgery time creates two forms of value: improving patient outcomes and reducing costs. First, shorter times under anesthetic and on cardiopulmonary bypass correlate with better surgical results ( Cheng *et al.*, 2018). Second, reducing healthcare costs brings broad societal benefits in both publicly and privately funded healthcare systems. Efficiency gains and reduced hospital costs may also lead to increased access to advanced medical care when coupled with corresponding policy and economic measures. This case raises important questions about how surgeons and the broader healthcare sector, which the World Health Organization (WHO) estimates to comprise 10% of global GDP ( World Health Organization, 2020), can become a leading market for AM services provided by makerspaces. We analyze the case as a latent business opportunity that has the potential to increase AM’s role in sustainable local production.

Our study follows Huarng’s (2013) work on business models, which proposed “a two-tier business model to assist entrepreneurship, consisting of a conceptual model (the first tier) for describing the business idea and a financial model (the second tier) for discovering the financial concerns” (p. 2102). We concentrate on the conceptual stage by documenting an innovation within the frame of a conceptual business model, leaving financial modeling to future work. Building on Cha’s (2020) work on global strategy and scholarship on micro-multinationals ( Czinkota, 2018; Gerbacia & Gerbacia, 2006; Stepanek, 2010), we indicate the value of developing a micro-multinational organization to leverage the value creating nodes most efficiently that include makerspaces, hospitals, and their supply chains. The case originated through the co-creation framework and collaborative processes led by some of the project participants, rather than it being a typical case study identified and then investigated by external researchers. Nevertheless, it is a valuable opportunity to expand the literature on user-driven technology in healthcare.

The article makes two contributions. First, it complements the literature on global strategy and innovation by highlighting how the makers’ organization process creates value through grassroots entrepreneurship. Second, it addresses the gap between innovation initiatives and their impact on business models. While the findings are focused on creating sustainable business models for makerspaces and other collaborative production sites, innovative startups, and multinational corporations may benefit from developing similar local collaborations to increase their strategic agility.

The article begins with a literature review before presenting the iProduce project’s approach to co-creation, open innovation, and makerspaces. The case presentation follows before a discussion about the processes and locations of value creation through AM in the medical sector. We explore AM’s potential to serve as a driver of local economic development and opportunities for makerspaces to create strong value propositions in other untapped training and educational fields. Further, we address how local entrepreneurship can address other production challenges by becoming a key resource in the local supply chain through distributed manufacturing.

## Literature review on co-creation, makerspaces, additive manufacturing, medical models, and points of collaboration

### Co-creation, open innovation, and makerspaces as sites of entrepreneurship

The iPRODUCE project centered strategies to achieve user involvement and collaboration from different stakeholders. Co-creation, co-production, and open innovation are all connected terms that have been widely used in the literature to describe different types of user involvement in various stages of the production process including research, developing and testing new ideas, and validating and testing products. Some terms, such as co-creation and co-production are often used as synonyms, with little differentiation between them ( Voorberg *et al.*, 2015).

Co-creation is a collaborative process that seeks to develop ideas and solutions in the context in which they emerge ( Puerari *et al.*, 2018; Sanders & Stappers, 2008). Co-production was promoted in the late 1970s as a framework to involve citizens in the production of public services ( Alford, 2014; Percy, 1978). More recently, the term co-production has been used to define a process where various stakeholders conceptualize and test solutions before they are implemented, or “an arrangement where citizens produce their own services at least in part” ( Brandsen & Pestoff, 2006, p. 6).

Another concept that directly relates to co-creation and co-production is open innovation (OI), understood as connecting internal research to outside ideas ( Chesbrough, 2004; Helfat, 2011). Open innovation principles cover “integrated collaboration, co-created shared value, cultivated innovation ecosystems, unleashed exponential technologies, and extraordinarily rapid adoption” ( Curley & Salmelin, 2013, p.2). Most importantly, product and service ideas are co-created with stakeholders that do not work at the company or organization that will bring them to market. Open innovation contrasts with closed innovation, by outsourcing and expanding the reach of where ideas originate ( Curley & Salmelin, 2013; Curley & Salmelin, 2018).

In recent years, open innovation initiatives have increased and are becoming more common within established industries. However, many of these innovation-focused activities are perceived as expensive, time-heavy, and require shifts in firm and employee outlook for the collaborative process to be effective ( Turkama & Kivikangas, 2016). While established industries often take more time to adapt and struggle with agility, start-ups, makerspaces, and user-driven initiatives are early adopters and do a degree of market testing using their own investments, fundraising campaigns, and accelerator programs.

Makerspaces and fablabs have expanded their presence within an open innovation framework. Makerspaces and fablabs are “open access communities for individuals to meet, socialize exchange ideas and work on projects related to technology, science and arts” ( Halbinger, 2018, p. 1) and are valuable spaces to support user driven innovation ( Neumeyer & Santos, 2019; Svensson & Hartmann, 2018). Makerspaces gained traction by becoming entrepreneurial sites, keeping their edge through the integration of current and upcoming technologies that address emerging market opportunities ( Hui & Gerber, 2017). While scholars identified the importance of user-driven initiatives in economic development ( van Holm, 2017), many makerspaces struggle to achieve their goal of transforming consumers into producers on a wide scale in the communities in which they are situated. Reasons contributing to this shortcoming include financing, management, local policies hindering development, poorly developed business models of creative spaces, and the perception of these spaces by community members where they are based. Nevertheless, the number of makerspaces and fablabs across the world increased in recent years ( Rosa *et al.*, 2017). They opened in libraries, schools, and even hospitals focusing on various digital manufacturing activities ( Abdullahi & Dewa, 2020; Uthmann, 2011).

### Additive manufacturing

Discussion of AM is commonly positioned within the proposed transition from industry 3.0 to 4.0 ( Da Xu *et al.*, 2018). In this conceptual framework, the new technologies of steam engines pushed the first industrial revolution, electricity and mass production drove the second industrial revolution, digitization and process refinement characterized the third industrial revolution, while “cyber-physical systems” are leading us toward a fourth industrial revolution ( Korner *et al.*, 2020). The term industry 4.0 grew out of a 2011 German government paper that set out goals for economic development ( Rojko, 2017) and captures the transition between the automation and information communication technologies that characterized industry 3.0 to the increased reliance on cyber-physical systems and decentralized production through additive manufacturing ( Dilberoglu *et al.*, 2017). Further work has identified shifts in interfirm networks “reorganizing consumption, manufacturing, innovation, and marketing” ( Achrol & Kotler, 2022).

Korner *et al.* (2020) conducted a systematic literature review on the “Integration of Additive Manufacturing and Industry 4.0.” One of their primary findings confirmed the relationship between AM and industry 4.0 that “points at AM as an enabler for redistributed manufacturing, because it can offer design freedom, on-demand production series for tooling, and the repair and refurbishment of metal parts” (p. 1061). They also identified a lack of research into the potential of redistributed manufacturing through AM to reduce supply chain and inventory costs. Savolainen and Collan (2020) found that “despite the technological buzz around AM and its potential groundbreaking effects to selected industries, the current engineering-focused literature runs short on contributions related to business-issues” (p. 2–3).

Bogers *et al.* (2016) is a helpful contribution to the business literature on AM presenting a case study of how a manufacturer of consumer goods could open its business model by utilizing AM to shift from a “manufacturer-centric to a consumer-centric value logic” (p. 225). The authors argued that AM creates the most value through consumer customization and the personalization of parts or products with limited manufacturing runs. This is accomplished by reducing or eliminating tooling costs and the time lag it creates in traditional reductive or mold-based manufacturing techniques. In doing so, the article presented a model for extending the benefits of AM beyond the prototyping and design process. However, one of the primary downsides of AM is the lack of scaling benefits, as AM is mostly suited for short production runs ( Hannibal & Knight, 2018). Reducing or eliminating tooling lowers design revision costs and time, but increased production volume does not reduce costs in AM.

The business model proposed by Bogers *et al.* (2016) builds on Zott and Amit's (2010) effort to identify “efficiency, complementarities, lock-in, and novelty to determine the value creation logic” (Bogers *et al.*, p. 227). A key distinction of this business model is that it is built from the position of a consumer goods manufacturer that may see AM as a direct threat to their business if consumers could simply print their own product. The model lays out a hybrid relationship between traditional and AM manufacturing with consumer lock-ins to the firm that is a supporting business model, rather than entirely replacing its traditional manufacturing and business formulations.

This business model informs entrepreneurship and innovation within makerspaces by pointing toward the strongest value proposition of AM in limited production runs and customization, the potential to significantly reduce transportation costs, to quickly address local demand, as well as one of the technology’s downsides in the lack of scaling benefits. This model also aligns with Hannibal & Knight’s (2018) suggestion that a key advantage of AM is reducing logistics costs if goods are manufactured closer to consumers, an aspect that aligns with our case.

### Bridging disciplines

The majority of literature on the medical applications for AM is technical and found in medical and engineering journals. This literature helps validate the technology that we discuss from a business perspective. It also identifies additional use cases for AM to drive innovation and better treatments in the healthcare sector, and the possibility for return of investment (ROI) for its development costs ( Marshall & McGrew, 2017; Su & Al’Aref, 2018; Svensson & Hartmann, 2018).

Culmone *et al.* (2019) identified the primary medical uses of AM as cranio-facial, dental, prosthetics (especially in developing countries), human tissue engineering, drug delivery systems, laboratory equipment, portable test tools, implants, surgical guides for screw insertions, and “anatomical models for both surgical planning and procedure training particularly in cases of rare pathologies'' (p. 461) This final use case is where our aortic aneurysm model is situated.

Gibson *et al.* (2006) evaluated the potential for rapid prototyping for medical applications in a period before the improvement and increased availability of 3D printers. The article highlighted some of the considerable technical limitations of rapid prototyping machines of the period, while recognizing the great potential of the technology. More recently, Bibb *et al.* (2015) joined the “fields of engineering and medicine” (p.2) in an applied study of design and prototyping, while Jones *et al.* (2016) evaluated the general feasibility of AM medical models. Salmi (2021) found that while the scientific research concentrated on AM created implants and bio manufacturing, there is increased interest in AM pre-operative models. Further, Salmi identified studies on the accuracy of AM medical models ( Ghomi *et al.*, 2021; Jockusch & Öxcan, 2020) and Singh and Ramakrishna’s (2017) imaging guidance for AM orthopedic implants. In 2017 with the latest update in 2020 ( United States Food and Drug Administration, 2020) the United State Food and Drug Administration (FDA) created a framework that ranges from guidance to regulation of AM devices in medicine. Preoperative medical models fall outside of current regulation.

Jones *et al.* (2016) examined the potential for the use of AM medical models in medical education to help clinicians better understand the anatomy of pathologic disease. The AM techniques used in this study are similar to the case presented in our article. Computed tomography (CT) and magnetic resonance (MR) images were imported into 3D modeling software before being manufactured through an AM extrusion process. This study included an example of a 3D printed aortic aneurysm with translucent, opaque, and black resin that could be viewed as a whole or separated into its constituent parts. The authors surveyed healthcare workers to gauge their view of their models. Results indicated that while healthcare workers valued the models, the participants noted the limitations of the models’ ability to represent real organs and tissue. Continuing, the authors found “the ability to produce multiple models of the same diseased organ allows the potential for more precise preoperative planning and practicing an operation both mentally and in a simulated manner” (p. 193–4). Findings specific to the heart model indicate “the aortic aneurysm model could potentially aid in selection of endograft, and relay concerns about proximity of the endograft to renal vessels” (p. 194). Salmi (2016) also contributed a detailed analysis of a heart created through AM. The author presented the case of a patient with “deformations and operations so surgeons feel that preoperative model would help them to plan the surgery before-hand and achieve better results” (p. 2). Technical details of the AM process can be found in the article, for our purposes, the finding that “preoperative models are mostly needed [in] cases where anatomy of the patient varies from normal” (Ibid., p.4) supports our findings. This case points to the limitations of existing traditionally manufactured models in representing the full breadth of the human form, especially idiosyncratic pathologies.

***Makerspaces and hospital collaborations.*** Makerspaces flourish when well-defined purpose meets technology capabilities. To achieve such a match, diverse types of professionals need to gain awareness of the existence of spaces with digital fabrication equipment, including additive manufacturing capabilities, and learn about the professional expertise available to provide support for imaginative experimentation across disciplines. Just this type of environment was created in Sweden.

In Sweden, six hospitals were equipped with six makerspaces supported by VINNOVA (The Swedish Innovation Agency). The purpose of this experimental intervention, as described by Svensson and Hartmann (2018), was to respond to a “formal request from the Swedish Ministry of Enterprise ‘to execute an initiative in order to increase the amount of cooperation-environments for the commercialization of innovations within the health and hospital sector’” (p. 277). The project received funding for three years between 2010 and 2013. The importance of this case, beyond the large number of new products and services developed in the period, is how it created value though its local, in situ aspects. Meaning that healthcare professionals in close contact with novel technologies experimented and prototyped their ideas emerging from cases in their daily medical work. The return on investment (ROI) was high. According to the authors “makerspaces, in the context of Swedish public hospitals, enable the development of innovations of a potential value that exceeds (by more than an order of magnitude) the requisite investment to establish and operate the makerspaces” ( Svensson & Hartmann, 2018, p. 284).

A similar project, on a smaller scale and within a different healthcare economy, came in the United States. The MakerNurse project at the University of Texas Medical Branch (UTMB Health) began in 2013 with the “goal of examining nurse innovation in U.S. hospitals and identifying tools and resources that could help more nurses bring their ideas to fruition and lead improvements in patient care” ( MakerNurse, 2022). This was the first makerspace built in a hospital in the United States. Nurses as key innovators of medical practices and devices have a long history. Gomez-Marquez and Young (2016) and Marshall and McGrew (2017) documented the role of nurses as innovators through the practice of “stealth making” (p. 56) that has gone on for years and shared in publications such as the American Journal of Nursing’s as “Practical Suggestions” (p. 57). Unfortunately, gender bias often devalued their contributions as unscientific and without merit.

Patients can also drive innovation in care. Sedini *et al.* (2021) document the case for patient innovation, facilitated through makerspaces that undergird their creative processes to reduce dropout and unrealized ideas. Prior EU Horizon 2020 grants and foundational support created infrastructure to support innovation, from initial idea formulation through to creating working prototypes, by addressing the tendency of potential innovations not making it past the ideation stage: “Makerspaces and Fab Labs can act, in connection with other relevant experts, to empower patients who own an innovative idea, providing them the ‘space for innovation’, composed by infrastructures, tools, methods, networks and money, thanks also to public or private fundings, which are more and more taking into consideration Patient Innovation as part of Responsible Research and Innovation domain” (Ibid., p. 4316).

These possible partnerships need to be explored from a business perspective by accounting for the resources necessary for long-term financially viable collaborations. The present article contributes an additional example of a successful innovation generated through makerspaces and hospitals when the collaboration was funded and facilitated by an outside grant agency. However, due to technological advancements, makerspaces do not need to be located inside of hospitals to facilitate innovation. Rather, existing makerspaces located near hospitals can offer valuable professional and educational services to the medical sector, if these services are visible and accessible within this sector and, arguably, also to local residents.

## Methods: the iPRODUCE project – interdisciplinary approaches and technology come together

The core goal of the iPRODUCE project ^1^ was to define a social manufacturing framework (SMF) to support open innovation and co-creation activities for the design, engineering, and production of consumer goods. The framework engaged stakeholders and interest groups on a local and Europe-wide level, including manufacturing enterprises (primarily SMEs and/or mid-caps), maker communities (including DIY, Fablab, makerspaces and startup communities), and general consumers. Stakeholders engaged in locally situated projects through co-creation tools to ensure that project solutions were driven by consumer needs.

The project comprised twenty partners, nine industries, five makerspaces/fablabs, and four research centers in France, Germany, Greece, Italy, and Spain. The partners were organized in the format of collaborative manufacturing demonstrator facilities (cMDF). Each cMDF is composed of SMEs, FabLab/makerspace, and a research partner. The local cMDFs all have manufacturing capabilities. The production capabilities (types of digital fabrication), involved in the project vary in scale and product focus within the consumer goods sector, from furniture to mobility. All cMDFs incorporated the user-driven and co-creation approach in their processes and local cases during the project regardless of product focus.

Key aims of the project were to identify opportunities for companies to learn from the makers’ approach and to challenge their production processes by creating replicable methods to collaborate with DIY communities on open innovation projects. These collaborative innovation networks ( Adner & Kapoor, 2010; Cha, 2020) may lead to viable and sustainable business opportunities lying dormant at their doorsteps. In this context, user-driven innovation, led by various makers and partners outside the consortium participated in the innovation process, challenging old ideas, and bringing fresh perspectives to meet consumer needs.

iProduce commenced in January 2020, just three months before the pandemic related lockdowns in Europe, which presented distinct challenges to executing the planned project action. The pandemic forced adaptation of the research project as it did in society and business. Sharma *et al.* (2020) and Donthu and Gustafsson (2020) wrote of the challenges facing businesses in the first wave of the pandemic, especially uncertainty, that reflect the challenges of managing a research project through this period. However, the uncertainty opened a unique opportunity for the project. A range of professionals from various fields were invited to the project meetings, many of them carried out online due to pandemic constraints, but nevertheless the meetings raised awareness across diverse sectors about digital fabrication and AM opportunities near them. This included healthcare professionals being brought into close contact with experts in digital manufacturing in several locations. A collaboration to design and produce personal protective equipment (PPE) grew out of these interactions. The stakeholders’ resources and skills allowed several of them to become part of the support system in their countries, producing PPE and other medical equipment to assist local hospitals and residents. This initiated broad collaborations between makerspaces and the medical sector to help meet the needs emerging from the pandemic.

While all makerspaces and fablabs in the project produced some type of PPE, each organization was distinct, fitting local idiosyncrasies. For example, in Spain, where the health system was overwhelmed early in the pandemic, DIY groups organized themselves online by sharing questions and designs through social media platforms ( Spanish Makers’, 2020). A number of external stakeholders joined the group of makers, which proved vital for its success. Healthcare professionals contributed the expertise required to identify their specific needs and advised on regulatory specifications for equipment, while makers brought knowledge of materials and manufacturing. This information exchange allowed for the quick creation of designs, models, and prototypes. After the testing and acceptance of the prototypes, makers started a larger production run on their 3D printers and organized distribution, which involved volunteers, taxi drivers, and even local police to distribute over one million face shields in Spain. The files for the products were shared widely under a Creative Commons (CC) license. A smaller scale collaboration also unfolded in Italy; the location of the case study presented in this article.

The iPRODUCE project conducted two rounds of business model workshops at each local cMDF. The first workshop in the Italian cMDF, was held in January 2021, before the case presented in this article had taken place. It explored value propositions within the consumer goods manufacturing sector aligned with the project description. The second workshop in May 2022, after the hospital case, revisited the business model value proposition and partnerships to accommodate novel market opportunities.

Huarng (2013) proposed a two-tier business model, split between the conceptual and financial models. The conceptual model covers the aspects of innovation (what), resource (how), market (who), and the value (why). The financial model covers cost, revenue, and profit. By addressing the importance of lowering the risk for entrepreneurship through internet based innovation, Huarng argued that the digital medium reduced the resources necessary, as it can be easily replicated, while also enabling easier market expansion due to its ‘ubiquity’ ( 2013, 2104).

Our methods initially concentrated on the conceptual perspectives of the business model by labelling the types of value created through the different cases explored by the cMDF and how these innovations could spur new business propositions. A case that grew from collaboration between the Italian cMDF and healthcare professionals early in the pandemic is presented in the following section.

## Results: The ProM (Prototyping Mechatronics facility) / Santa Chiara Hospital aortic aneurysm prosthesis collaboration case

In 2021, physicians at the Santa Chiara Hospital in Trento, Italy diagnosed a patient with an aortic aneurysm, a dilation or swelling of a portion of the aorta, the largest artery in the human body. This is a life-threatening condition. The standard treatment is surgery to insert a stent, a retractor, or a custom-made prosthesis. This is a delicate operation, and each patient has a unique clinical case and anatomy. To prepare for the surgery, the patient underwent MRI and CT scans for a precise diagnosis.

A member of the medical team had engaged with the local makerspace during the height of the pandemic on a PPE-related project and suggested the possibility of using the scans to print an aorta model to help the surgeons prepare for the operation. They contacted the ProM Facility laboratory of Trentino Sviluppo in Polo Meccatronica and commissioned a model of the aortic aneurysm to be manufactured based on the patient’s CT data provided by the hospital's vascular surgery unit.

ProM engineers began with scan data for a complete anatomical model of the patient’s rib cage area from which they isolated the section of aorta with the aneurysm to create a 3D printable file. The engineers used the software package nTopology to rework the full chest scan data into a model of the area of concern. Their design work resulted in an accurate computer model of the internal and external anatomy of the aorta. The model was 3D printed out of TPU (thermoplastic polyurethane) with variable thickness from 0.4 mm to 0.8 mm.

**Image 1.**
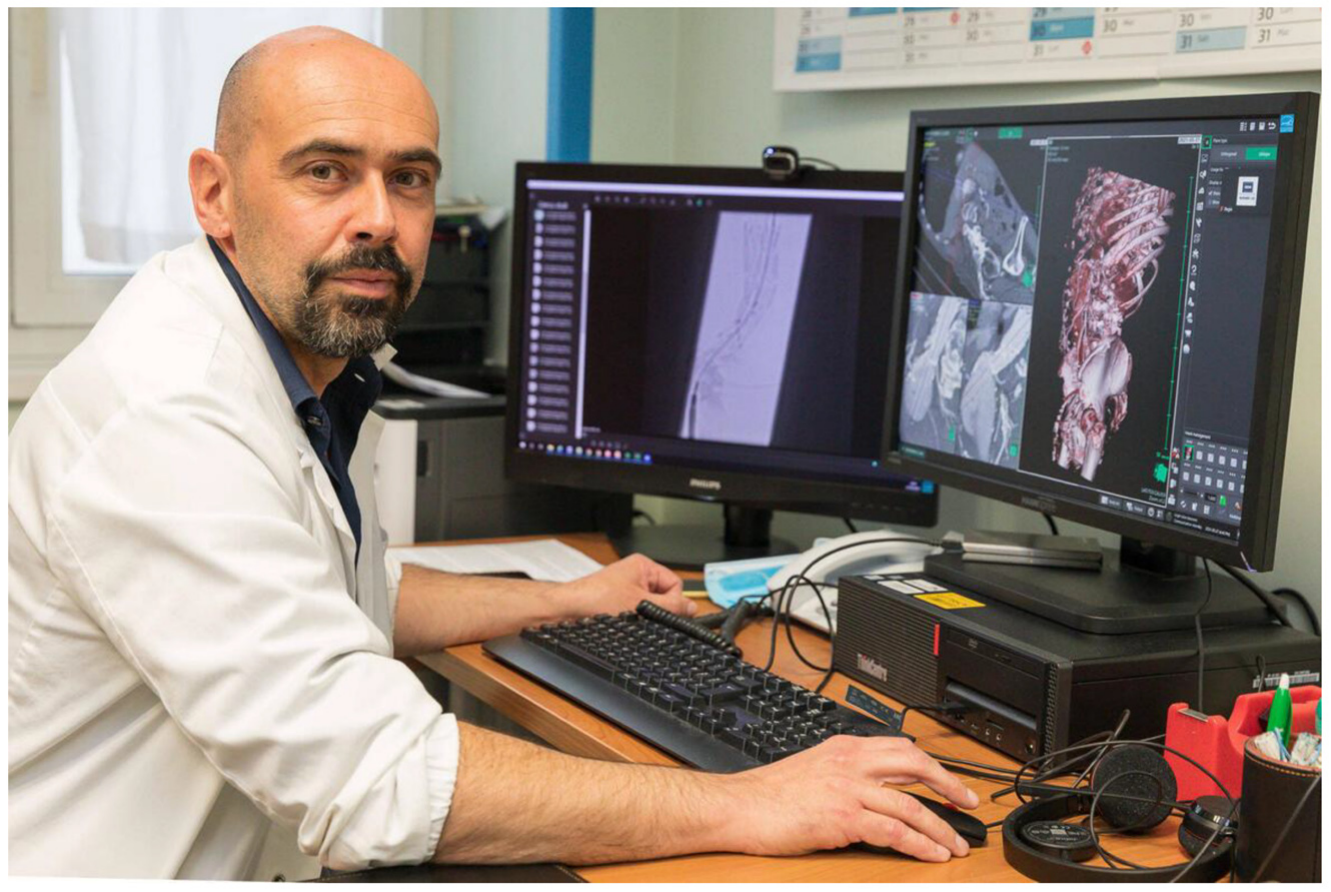
Stefano Bonvini with a comparison between the raw models as extracted from CT data and the final model ready for printing ( Intervento all’aorta, 2021).

Stefano Bonvini ( Image 1), director of vascular surgery at Santa Chiara hospital mentioned that:

“In some cases the anatomy of a patient does not allow us to have a precise picture of the possibilities of success of the operation. In these cases, having the possibility to simulate the starting conditions is a great advantage, proving in advance the effectiveness of the various maneuvers. We can evaluate the feasibility of the intervention or even foresee some variables, thus reducing the necessary maneuvers and therefore the times and costs of the procedure, avoiding useless attempts” ( Intervento all’aorta, 2021).

**Image 2.**
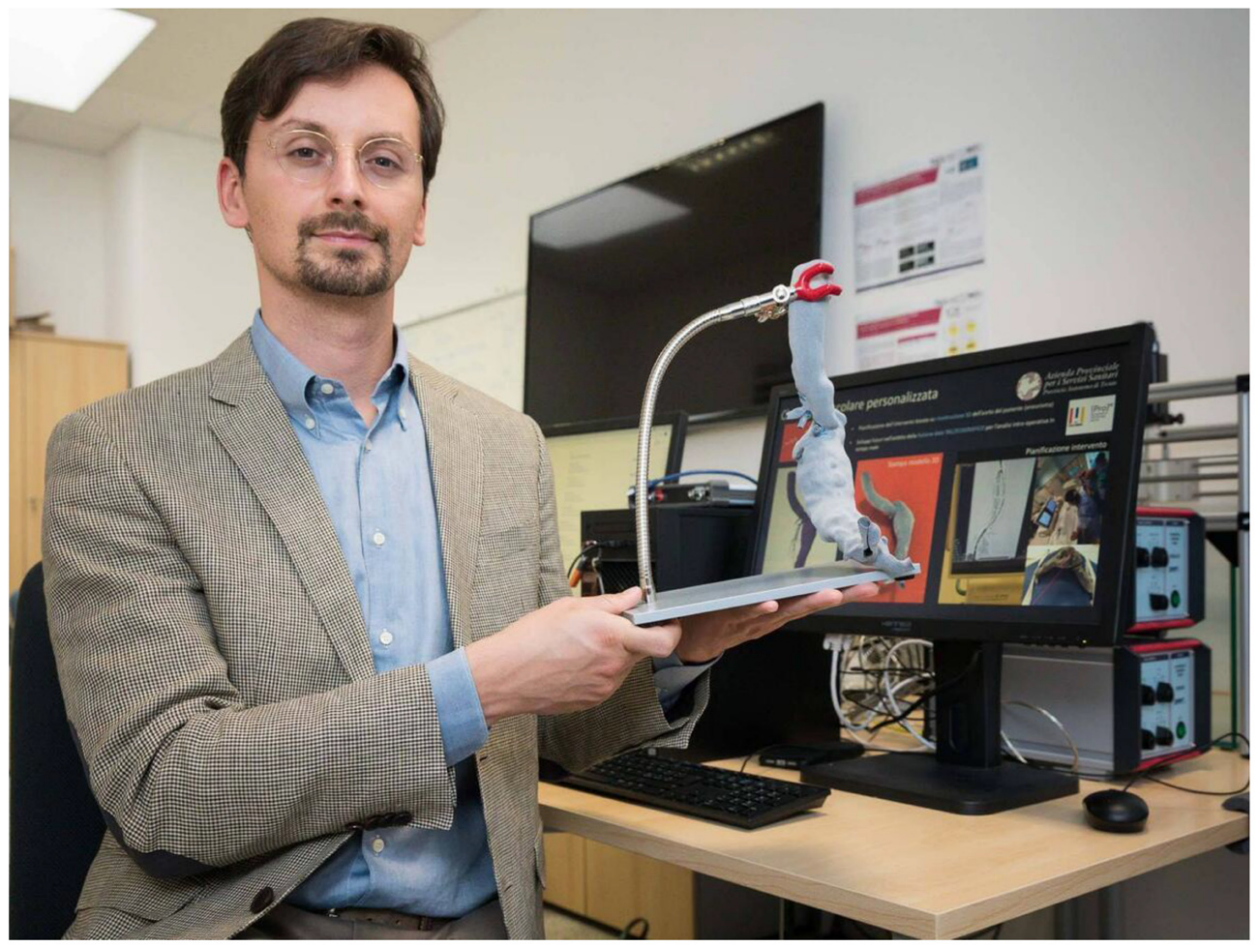
Libertario Demi with the printed aortic aneurysm mounted on a stand and ready to be inspected and tested by the medical team ( Intervento all’aorta, 2021).

Libertario Demi ( Image 2), professor of electronic and computer bioengineering and head of the Ultrasound Lab Trento (ULTRa) at the University of Trento, believed “This model, on a 1 to 1 scale, could also be useful for training activities for new doctors, because it will allow them to learn the techniques of intervention on non-simple vessel structures” ( Intervento all’aorta, 2021).

The printed aortic aneurysm had a high degree of accuracy. Additionally, the elasticity of TPU material used to print the replica aortic aneurysm behaved similarly to human tissue and its density allowed a clear ultrasound analysis during preoperative simulation. While this is not the first 3D printed aorta, the properties of the material – radiopaque and transparent – and its short production time are of interest to surgical teams. The outcome of this case is remarkable, as the successful collaboration reduced surgery time by 70%, which correlate with better patient outcomes ( Cheng *et al.*, 2018).

## Discussion and analysis: makerspaces as sites of innovation

AM and local user-innovation spaces are commonly seen as untapped resources because that have not created the job growth, product innovations, and collaborations as promised by their boosters. In recent years, the EU, as part of its HORIZON 2020 program, has opened calls for projects that would explore local production opportunities and facilitate knowledge exchange among local manufacturing resources in makerspaces and fablabs with established businesses. The iProduce project set out to intentionally foster collaborations that often fail to organically materialize. This business opportunity evolved out of these collaborations, though not as initially intended. As noted, the coronavirus disease (COVID-19) pandemic forced shifts in this project and participants responded by producing PPE. A member of the medical team at the hospital remembered their interaction with the AM facility and sought out their capabilities.

The adaptation of the iProduce project through the COVID-19 pandemic and the example of the collaboration that led to the 3D printed aortic aneurysm offer important lessons for businesses of all sizes looking to increase their strategic agility to capture new forms of value that draw on their existing strengths. Strategic agility literature focuses on three primary vectors of this concept: “IT agility, supply chain agility (SCAGI) and agile and sustainable productions” ( Shams *et al.*, 2021). This case provides insight across all three focal points.

Business model innovation is often born out of necessity. Andries *et al.* (2013) researched how uncertainty pushed experimentation in business models though a “combination of action and planning results not only in substantial variety, but it can also be organized in a cost-effective manner and, therefore, it offers good prospects for the survival and growth of entrepreneurial ventures operating under uncertainty” (p. 307). Furthermore, Klein *et al.* (2021) examined the connection between sustainability and business model innovation, finding that “firms with a commitment to sustainability do not ‘automatically’ innovate their business models.… sustainability is a driver to deepen technology-oriented strategic behavior, market-focused information gathering and entrepreneurial behavior, which subsequently lead to the BM's innovation” (p. 283). Returning to the discussion of strategic agility, the makerspace/medical model opportunity was discovered through an experiment and captures value through the three vectors of strategic ability of “IT agility, supply chain agility (SCAGI) and agile and sustainable productions” ( Shams *et al.*, 2021).

Chebbi *et al.* (2017) centered innovation within the process of the internationalization of firms and presented findings on how firms “can help develop a mutual knowledge transfer between heterogeneous local actors such as acquired firms and own subsidiaries” (p. 132). Large firms in the process of internationalizing can learn from the collaborative, user-driven innovation model that produced the 3D aorta model. Rather than looking to orchestrate efficiently through a top-down internationalization process within an organization, firms may create more value by empowering individual units to develop user-driven initiatives. This finding fits within the bottom-up paradigm of global value-chain literature identified by De Marchi *et al.* (2020).

In line with our findings and broader review of AM literature, Hennelly *et al.* (2019) examined the scalability of production in makerspaces and argued that “It seems unlikely that makerspaces will take root in industries or industry segments characterized by long production runs and/or industry segments where manufacturing is already highly automated” (p. 550). Indeed, the proposed makerspace/medical model business discussed falls into the highly curated and bespoke model making through low volume production runs.

Recent research demonstrates that user-driven initiatives such as the ones encountered in makerspaces can be great catalysts for both innovation and collaboration. Some governments have supported these types of initiatives focusing on both economic and social benefits through targeted policies ( Halbinger, 2018). In the medical sector, the demands for user-driven innovation range from casts and braces to the development of tools and props that address surgical and training challenges. Demand is clear and the model we propose is one way to leverage existing resources and institutions to meet it by creating sustainable local businesses with great social impact. Meeting this demand could help makerspaces live up to their promise as Halbinger (2018) noted “Governments may find that investments in supporting user innovation via infrastructures like makerspaces may well pay off in economic benefits to society in the form of increases in the production of economically valuable innovations and a vehicle to overcome its under-diffusion” (p. 2035).

While not conclusive, research suggests the potential for entrepreneurial makerspaces to contribute to their communities ( Hennelly *et al.*, 2019). The potential of their contributions may be far-reaching, supporting sustainable local economic development. Hui and Gerber (2017) described how “makerspaces support entrepreneurial skill development, such as using makerspace technologies to build and market new products, and self-efficacy development, such [as] having the confidence to develop new ventures” (p. 2035). They continued, describing how to develop just this sort of space: “Developing an entrepreneurial makerspace goes beyond inviting people with entrepreneurial goals. It involves creating opportunities offline and online to develop skills and self-efficacy in a range of entrepreneurship tasks, from manufacturing to marketing” ( Hui & Gerber, 2017, p. 2028). While noting that more research is needed towards digital innovation and fabrication networks (DIFN), Seo-Zindy and Heeks (2017) wrote “While it is questionable whether DIFNs would ever be able to democratize and revolutionize the global economy, empirical findings do suggest a shift in production models” (p. 13).

The iProduce project and the business opportunity described fit well within Smith and Light’s (2017) observations of makerspaces: “Our concluding point is merely to draw attention to the fact that this does not happen automatically” (p. 173). While it does not represent a commodity mass-produced through local and distributed production, it may provide sustainable income for makerspaces, lower healthcare costs, and save lives by creating cross-international opportunities for local production. In the case of this project and the resulting business opportunity presented in this paper, the “liability of smallness” coincided with the “liability of newness” as discussed by Eggers (2020), but the flexibility of participants led to opportunity. Training professionals with 3D printed models is a business opportunity with a potentially strong value proposition for makerspaces with expertise in AM.

### Directions for future research: an AM micro-multinational

The 3D printed aortic aneurysm is evidence for the potential of AM to meaningfully contribute to surgeons’ pre-operative preparations for patients with idiosyncratic conditions and more broadly in the training of medical professionals. The case presented several opportunities for collaboration between makerspaces and the healthcare sector through AM. Healthcare professionals face high demands from their jobs, while makerspaces are a largely underutilized resource. The partnership in the case generated value through makers increasing their knowledge of the medical field and AM professionals sharing their expertise with healthcare professionals about emerging materials and technologies that can meet their needs.

The case leads to a reevaluation of the business models underpinning makerspaces, which deserve further research. Many are fully publicly funded, some have hybrid funding models connected to startup ecosystems, and others are fully privately or participant funded. Privately funded and hybrid funded makerspaces primarily function as local SMEs dependent on both niche and global markets, a fact highlighted by the COVID-19 pandemic and related global supply chain pressure. Despite having local foci and providing local services, the pandemic increased financial pressure on makerspaces and their need to create durable business models to achieve lasting financial viability. Deploying AM resources to capture value in the medical sector is a strong business opportunity for makerspaces to live up to their promise as sites of collaborative innovation. This business opportunity also aligns with environmental sustainability goals through the corresponding choice of materials and shifting supply chains toward local production.

A potential topic for future research is on the potential for AM to underpin micro-multinational startups anchored by makerspaces. Micro-multinationals, as a business ecosystem ( Cha, 2020), generally leverage technology to tap global business opportunities from their inception ( Gerbacia & Gerbacia, 2006; Mettler & Williams, 2011). Makerspaces and fablabs are strong candidates to create a micro-multinational as it aligns with their need to expand their sources of income beyond public funding. It may also open a way for makerspaces to increase their footprint in the local production mesh through social manufacturing. Makerspaces and fablabs serving as the lead contact in their location efficiently utilizes resources and hospitals, universities, etc. benefit from interlocal agreements.

One example of how a micro-multinational could reshape markets is to store high-definition CT and MRI scans used to produce high-definition medical models with patient consent in global digital medical database available for export and printing. Managing the repository as a micro-multinational through a network of makerspaces may provide optimize flexibility and value capture. Translating case descriptions into various languages would increase its global reach ( Stepanek, 2010). A database such as this is highly relevant to healthcare professionals and instructors, enabling them to search for both routine and idiosyncratic cases to assist diagnosis, treatment, and training. Micro-multinationals have low startup costs and capture value by deploying modern IT infrastructure from their inception. Exploring the potential of a micro-multinational organized as an affiliate network, rather than the outsourcing described by Gerbacia and Gerbacia (2006) is worthy of further research.

Another direction of future research may be to examine AM and makerspaces from a knowledge spillover theory of entrepreneurship (KTSE) perspective. Audretsch *et al.* (2021) examined KTSE and the broader literature on entrepreneurship to draw linkages “to open innovation and knowledge transfer research using various origins of knowledge spillover to predict innovation performance.”

## Conclusions

The findings presented in this article emerged from collaborations linked to the iPRODUCE Horizon 2020 project. The case presented follows the project purpose, which has the primary aim of delivering a novel social manufacturing platform to enable multi-stakeholder interactions and collaborations to support user-driven open-innovation and co-creation. This case illustrates that by having shared purposes, an applied co-creation approach, and open innovation process, a mesh of fablabs and makerspaces can function as a large, distributed manufacturer using local resources, reducing transportation costs and environmental impact, leading to a paradigm shift in production. AM can realize its full potential and disruptive possibilities when matched with a supporting business model. In the case study presented, makerspaces and fablabs have the potential to partner with the medical field to capture value by joining their expertise and manufacturing equipment with the needs of nearby hospitals to create sustainable local business growth organized through micro-multinationals and facilitated by open innovation. Such partnerships will leverage IT advances, shift supply chains, and the organization of production into nimble, strategically agile businesses.

## Data Availability

The data for this article consists of bibliographic references, which are included in the References section.
