# Supplementary material for: The Beating Heart of Untapped Business Opportunities for Additive Manufacturing
Source: Open Res Eur. 2023 Sep 7;3:143. [Version 1] doi: 10.12688/openreseurope.16270.1 (PMC11226946; doi:10.12688/openreseurope.16270.1)
Supplement: Peer review history

# Reviewer report: Reviewer response for version 1

**DOI:** 10.21956/openreseurope.17565.r37937

**Published:** 2024-03-12

**Authors:** Leonardo A. García-García

**Affiliations:** 1 School of Engineering and Informatics, University of Sussex, Brighton, England, UK

The paper aims to present a case on the use of Additive Manufacturing  for the generation of pre-operative 3D models. The manuscript presents also a detailed limited number of cases and discusses their application.

**Comments:**

 P3: the methodology to be followed needs to be described briefly at the end of the of the introduction,

**Literature Review:**

 Needs to discuss drawbacks of AM.

 Is it true that AM gives freedom for design and implementation, but also its application is limited in most cases as prototypes?

 P5 Jones et al.:  reword the paragraph, it must be improved to make sure that the authors are still talking about Jones et al. What are the main findings?

 Do healthcare practitioners welcome the technology, what has to be improved to have a full acceptance? 

 Before methods, the Projects need to present information to the cases found, mention specific innovation cases, what were the main outcomes and the real impact they had had in the healthcare sector. 

 The methods section must not include the results, but rather talk about what you will evaluate, what are the parameters observed to have a successful case, authors must mention what information will they be considering, i, e. number of cases analysed, which countries, places, projects, number of projects per year, successful cases, partnerships, etc.

 In the case of the iProject, must consider discussing about the same, but focused on the project, you must discuss that you will analyse countries cases, network, collaboration, etc. 

**Results:**

 In the results, must include the main outcomes of all the cases analysed and presented. Information that will support the case of this business 

 Figure 1, I would recommend to add images of the cases.

 Also: image 2, focusing on the model is more relevant and will provide a good support to the case. Provide images of the outcomes analysed rather than of persons unless the images of the person is relevant to the manuscript. 

 In general, the paper does not have a clear division among introduction, methods and results. This is because all the sections describe successful cases. This is a drawback because it is not clear what is the methodology followed and there is no clear descriptions of the results. 

 Discussion has a very good structure, but it can be improved on explaining some negative findings form the cases analysed, i.e. COVID impacted the business, healthcare practitioners need to travel, etc. also drawback on the use of AM. The discussion must include information of what other cases are available but not included in the analysis, mention and discuss why it has not been a successful business or what did you find in the literature that is making difficult to implement this BM. Explain the difficulties you found in the cases analysed and why this happened. 

 P9 paragraph 2. here you mention three primary vectors to increase the strategic agility. Are these important parameters that must be analysed in each case? 

 Ref in Page 7, must be be 2014 not 2104.

 References are good, but some of these are more than 10 years old.

Is the case presented with sufficient detail to be useful for teaching or other practitioners?

Partly

Is the work clearly and accurately presented and does it cite the current literature?

Yes

If applicable, is the statistical analysis and its interpretation appropriate?

Not applicable

Are all the source data underlying the results available to ensure full reproducibility?

No source data required

Are the conclusions drawn adequately supported by the results?

No

Is the background of the case’s history and progression described in sufficient detail?

Yes

Reviewer Expertise:

Additive manufacturing, 3D modelling, Medical devices for rehabilitation, wearable electronics, smart textiles, Biomechanics, development of orthoses.

I confirm that I have read this submission and believe that I have an appropriate level of expertise to confirm that it is of an acceptable scientific standard, however I have significant reservations, as outlined above.

# Response

**Published:** 2024-05-01

**Authors:** Isabel Froes

Response to reviewer 2:   Firstly, we’d like to thank the reviewer for the time and helpful reviews, which we believe have helped us improve our work. All the comments are presented in the following with their respective responses. **Comments:**

  

1. P3: the methodology to be followed needs to be described briefly at the end of the of the introduction,

  We’ve added a short description of our methods to the Introduction. **Literature Review:**

1. Needs to discuss drawbacks of AM.
  Is it true that AM gives freedom for design and implementation, but also its application is limited in most cases as prototypes?

  To address R2’s concerns about a lack of discussion of the drawbacks of AM, we added discussion of the limitations of AM for mass production, while recognizing that the case documented in our study focuses on bespoke, individualized manufacturing of medical models. That is, it does not require mass production of uniform products, thus making it ideal for AM. Our discussion is through Bogers et al. (2016), Lemu (2018), Hannibal & Knight (2018), and Jung et al. (2023).  

1. P5 Jones et al.:  reword the paragraph, it must be improved to make sure that the authors are still talking about Jones et al. What are the main findings?
  Do healthcare practitioners welcome the technology, what has to be improved to have a full acceptance?

  We clarified our discussion of Jones et al. (2016) and included their survey findings that document the favorable views of their models by respondents. 

  

1. Before methods, the Projects need to present information to the cases found, mention specific innovation cases, what were the main outcomes and the real impact they had had in the healthcare sector.

  We reorganized the project presentation by moving it into the Methods section. Our participation in the project enabled our research. The cases have been relocated in the now rewritten Results section .  

1. The methods section must not include the results, but rather talk about what you will evaluate, what are the parameters observed to have a successful case, authors must mention what information will they be considering, i, e. number of cases analysed, which countries, places, projects, number of projects per year, successful cases, partnerships, etc. In the case of the iProject, must consider discussing about the same, but focused on the project, you must discuss that you will analyse countries cases, network, collaboration, etc.

  We’ve fully updated and rewrote the Methods and Results section, and added the additional information requested. In the revised Results section we present the two case studies under consideration including their location, network, and the form of the collaboration that took place. **Results:**

1. In the results, must include the main outcomes of all the cases analysed and presented. Information that will support the case of this business 

  In the revised Results section we present the outcome of both case studies under consideration.

  

1. Figure 1, I would recommend to add images of the cases.

  Figure 1 and Figure 2 were images of the second case study in the submitted draft. This case is discussed in more detail and the images illustrate the work that was done.

  

1. Also: image 2, focusing on the model is more relevant and will provide a good support to the case. Provide images of the outcomes analysed rather than of persons unless the images of the person is relevant to the manuscript. 

  Thank you for noting this. In this case, the person in is holding the model as a reference scale for viewers.

  

1. In general, the paper does not have a clear division among introduction, methods and results. This is because all the sections describe successful cases. This is a drawback because it is not clear what is the methodology followed and there is no clear descriptions of the results. 

These points are well taken. We have reorganized the article to more clearly introduce our investigation, delineate our methods, before then presenting the case studies in the results before our discussion.

  

1. Discussion has a very good structure, but it can be improved on explaining some negative findings form the cases analysed, i.e. COVID impacted the business, healthcare practitioners need to travel, etc. also drawback on the use of AM. The discussion must include information of what other cases are available but not included in the analysis, mention and discuss why it has not been a successful business or what did you find in the literature that is making difficult to implement this BM. Explain the difficulties you found in the cases analysed and why this happened. 

  We appreciate this comment and the suggestions. The Results and Discussion now includes challenges encountered. In fact, both case studies emerged in the scope of the covid pandemic. The first case study directly adapted the makers orientation to address the need for PPE. The second case study happened because of maker / hospital collaborations during covid that created the connection out of which the heart model was created. Within the EU Innovation Action that led to these two cases studies, the numerous interactions and outcomes are beyond the scope of a single article, however we see opportunities to explore such suggestions in future research.    

1. P9 paragraph 2. here you mention three primary vectors to increase the strategic agility. Are these important parameters that must be analysed in each case? 

  Indeed. The three vectors are what strategic agility scholars identify and should be set against out two cases. We’ve added note of this, and more broadly for developing novel business models, especially micro-multinationals, which we focus on in our Discussion with revisions.

  

1. Ref in Page 7, must be be 2014 not 2104.

  Thank you for nothing this reference typo. This point has been addressed.

  

1. References are good, but some of these are more than 10 years old.

  Thank you. We’ve revised our literature and added additional current references to further illustrate and contextualise the state of the field.

# Response

**Published:** 2024-05-27

**Authors:** Isabel Froes

Firstly, we’d like to thank the reviewer for the time and helpful reviews, which we believe have helped us improve our work. All the comments are presented in the following with their respective responses. **Comments:**

  

1. P3: the methodology to be followed needs to be described briefly at the end of the of the introduction,

We’ve added a short description of our methods to the Introduction. **Literature Review:**

1. Needs to discuss drawbacks of AM.
  Is it true that AM gives freedom for design and implementation, but also its application is limited in most cases as prototypes?

To address R2’s concerns about a lack of discussion of the drawbacks of AM, we added discussion of the limitations of AM for mass production, while recognizing that the case documented in our study focuses on bespoke, individualized manufacturing of medical models. That is, it does not require mass production of uniform products, thus making it ideal for AM. Our discussion is through Bogers et al. (2016), Lemu (2018), Hannibal & Knight (2018), and Jung et al. (2023).  

1. P5 Jones et al.:  reword the paragraph, it must be improved to make sure that the authors are still talking about Jones et al. What are the main findings?
  Do healthcare practitioners welcome the technology, what has to be improved to have a full acceptance?

We clarified our discussion of Jones et al. (2016) and included their survey findings that document the favorable views of their models by respondents. 

  

1. Before methods, the Projects need to present information to the cases found, mention specific innovation cases, what were the main outcomes and the real impact they had had in the healthcare sector.

We reorganized the project presentation by moving it into the Methods section. Our participation in the project enabled our research. The cases have been relocated in the now rewritten Results section .  

1. The methods section must not include the results, but rather talk about what you will evaluate, what are the parameters observed to have a successful case, authors must mention what information will they be considering, i, e. number of cases analysed, which countries, places, projects, number of projects per year, successful cases, partnerships, etc. In the case of the iProject, must consider discussing about the same, but focused on the project, you must discuss that you will analyse countries cases, network, collaboration, etc.

We’ve fully updated and rewrote the Methods and Results section, and added the additional information requested. In the revised Results section we present the two case studies under consideration including their location, network, and the form of the collaboration that took place. **Results:**

1. In the results, must include the main outcomes of all the cases analysed and presented. Information that will support the case of this business 

In the revised Results section we present the outcome of both case studies under consideration.

  

1. Figure 1, I would recommend to add images of the cases.

Figure 1 and Figure 2 were images of the second case study in the submitted draft. This case is discussed in more detail and the images illustrate the work that was done.

  

1. Also: image 2, focusing on the model is more relevant and will provide a good support to the case. Provide images of the outcomes analysed rather than of persons unless the images of the person is relevant to the manuscript. 

Thank you for noting this. In this case, the person in is holding the model as a reference scale for viewers.

  

1. In general, the paper does not have a clear division among introduction, methods and results. This is because all the sections describe successful cases. This is a drawback because it is not clear what is the methodology followed and there is no clear descriptions of the results. 

These points are well taken. We have reorganized the article to more clearly introduce our investigation, delineate our methods, before then presenting the case studies in the results before our discussion.

  

1. Discussion has a very good structure, but it can be improved on explaining some negative findings form the cases analysed, i.e. COVID impacted the business, healthcare practitioners need to travel, etc. also drawback on the use of AM. The discussion must include information of what other cases are available but not included in the analysis, mention and discuss why it has not been a successful business or what did you find in the literature that is making difficult to implement this BM. Explain the difficulties you found in the cases analysed and why this happened. 

We appreciate this comment and the suggestions. The Results and Discussion now includes challenges encountered. In fact, both case studies emerged in the scope of the covid pandemic. The first case study directly adapted the makers orientation to address the need for PPE. The second case study happened because of maker / hospital collaborations during covid that created the connection out of which the heart model was created. Within the EU Innovation Action that led to these two cases studies, the numerous interactions and outcomes are beyond the scope of a single article, however we see opportunities to explore such suggestions in future research.

1. P9 paragraph 2. here you mention three primary vectors to increase the strategic agility. Are these important parameters that must be analysed in each case? 

Indeed. The three vectors are what strategic agility scholars identify and should be set against out two cases. We’ve added note of this, and more broadly for developing novel business models, especially micro-multinationals, which we focus on in our Discussion with revisions.

  

1. Ref in Page 7, must be be 2014 not 2104.

Thank you for nothing this reference typo. This point has been addressed.

  

1. References are good, but some of these are more than 10 years old.

Thank you. We’ve revised our literature and added additional current references to further illustrate and contextualise the state of the field.

# Reviewer report: Reviewer response for version 1

**DOI:** 10.21956/openreseurope.17565.r37940

**Published:** 2024-02-20

**Authors:** Kasin Ransikarbum

**Affiliations:** 1 Ubon Ratchathani University, Mueang Si Khai, Ubon Ratchathani, Thailand

1. Introduction: Suggest that the authors consult more business model and supply chain papers related to AM. In addition, more recent papers related to AM healthcare model in other regional case studies should be added.
2. Introduction: Suggest that trend data relevant to AM marketplaces for recent years are added. There are also both profit-oriented and non-profit oriented AM marketplaces, which should also be discussed.
3. Section 2: “Nevertheless, the number of makerspaces and fablabs across the world increased in recent years (Rosa et al., 2017).” Some sentences are not convincing here. That is, the authors claim that the number of makerspaces increasing recently but cite the 2017 papers. Supporting number for 2020 or more (recent 5 years) is better.
4. “Reducing or eliminating tooling lowers design revision costs and time, but increased production volume does not reduce costs in AM.” This sentence seems to need a proper citation. 
5. Section “Bridging disciplines”  Recent papers are better here. Citing other regional case studies may also support the global perspective. 
6. It would be also interesting to share interview/discussion results from end users who use AM in their healthcare and/or business view. That is, suggest that a subsection contributing to remarks from healthcare professionals and business owners are added for managerial implications.  

Is the case presented with sufficient detail to be useful for teaching or other practitioners?

Partly

Is the work clearly and accurately presented and does it cite the current literature?

Partly

If applicable, is the statistical analysis and its interpretation appropriate?

Partly

Are all the source data underlying the results available to ensure full reproducibility?

Yes

Are the conclusions drawn adequately supported by the results?

Partly

Is the background of the case’s history and progression described in sufficient detail?

Yes

Reviewer Expertise:

Logistics and Supply Chain Management; Decision Support System Development; Additive Manufacturing

I confirm that I have read this submission and believe that I have an appropriate level of expertise to confirm that it is of an acceptable scientific standard, however I have significant reservations, as outlined above.

# Response

**Published:** 2024-05-01

**Authors:** Isabel Froes

**Response to Reviewer 1:** Firstly, we’d like to thank the reviewer for the time and helpful reviews, which we believe have helped us improve our work. All the comments are presented in the following with their respective responses.

1. **Introduction:** Suggest that the authors consult more business model and supply chain papers related to AM. In addition, more recent papers related to AM healthcare model in other regional case studies should be added. We both engaged further recent research on AM BMs and specific to the healthcare sector. We believe that these additions fit better in the literature review rather than the introduction. The additional references are: Lemu (2018), Jung et al. (2023), Open Source Medical Supplies (n.d.), Osterwalder A & Pigneur Y. (2010), Pasirayi, S., & Richards, T. J. (2023), and Pereira, T., Kennedy, J. V., & Potgieter, J. (2019).
2. **Introduction:** Suggest that trend data relevant to AM marketplaces for recent years are added. There are also both profit-oriented and non-profit oriented AM marketplaces, which should also be discussed.

We’ve added trend data for the AM industry as a whole from an industry report to the literature section on AM and business models. Unfortunately, we were not able to find reliable trend data for AM marketplaces in our research. If the reviewer knows of such data, we would very much appreciate and would thank in advance for a reference.

1. **Section 2: **“Nevertheless, the number of makerspaces and fablabs across the world increased in recent years (Rosa et al., 2017).” Some sentences are not convincing here. That is, the authors claim that the number of makerspaces increasing recently but cite the 2017 papers. Supporting number for 2020 or more (recent 5 years) is better.

We’ve added the latest data from Distributed Design Market Platform, n.d. We believe this is current and supports our suggestion of an increase.

1. **“Reducing or eliminating** tooling lowers design revision costs and time, but increased production volume does not reduce costs in AM.” This sentence seems to need a proper citation. 

We’ve added reference to Pereira et al., 2019 to support this statement.  

1. **Section “Bridging disciplines**”  Recent papers are better here. Citing other regional case studies may also support the global perspective. 

We’ve revised this section, and it references case studies in Europe and the United States. The following section on makerspace and hospital collaborations also includes multinational perspectives.  

1. It would be also interesting to share interview/discussion results from end users who use AM in their healthcare and/or business view. That is, suggest that a subsection contributing to remarks from healthcare professionals and business owners are added for managerial implications.

We agree that conducting additional interviews to gain additional insights into the potential collaborations between makerspaces and healthcare professionals is an interesting line of research. We have added this point as a suggestion for future research in the end of the article, as we believe such research could standalone as new article in the near future.

# Response

**Published:** 2024-05-27

**Authors:** Isabel Froes

Firstly, we’d like to thank the reviewer for the time and helpful reviews, which we believe have helped us improve our work. All the comments are presented in the following with their respective responses. **Introduction:** Suggest that the authors consult more business model and supply chain papers related to AM. In addition, more recent papers related to AM healthcare model in other regional case studies should be added. We both engaged further recent research on AM BMs and specific to the healthcare sector. We believe that these additions fit better in the literature review rather than the introduction. The additional references are: Lemu (2019), Jung et al. (2023), Open Source Medical Supplies (n.d.), Osterwalder A & Pigneur Y. (2010), Pasirayi, S., & Richards, T. J. (2023), and Pereira, T., Kennedy, J. V., & Potgieter, J. (2019).

1. **Introduction:** Suggest that trend data relevant to AM marketplaces for recent years are added. There are also both profit-oriented and non-profit oriented AM marketplaces, which should also be discussed.

We’ve added trend data for the AM industry as a whole from an industry report to the literature section on AM and business models. Unfortunately, we were not able to find reliable trend data for AM marketplaces in our research. If the reviewer knows of such data, we would very much appreciate and would thank in advance for a reference.

1. **Section 2: **“Nevertheless, the number of makerspaces and fablabs across the world increased in recent years (Rosa et al., 2017).” Some sentences are not convincing here. That is, the authors claim that the number of makerspaces increasing recently but cite the 2017 papers. Supporting number for 2020 or more (recent 5 years) is better.

We’ve added the latest data from Distributed Design Market Platform, n.d. We believe this is current and supports our suggestion of an increase.

1. **“Reducing or eliminating** tooling lowers design revision costs and time, but increased production volume does not reduce costs in AM.” This sentence seems to need a proper citation. 

We’ve added reference to Pereira et al., 2019 to support this statement.  

1. **Section “Bridging disciplines**”  Recent papers are better here. Citing other regional case studies may also support the global perspective. 

We’ve revised this section, and it references case studies in Europe and the United States. The following section on makerspace and hospital collaborations also includes multinational perspectives.  

1. It would be also interesting to share interview/discussion results from end users who use AM in their healthcare and/or business view. That is, suggest that a subsection contributing to remarks from healthcare professionals and business owners are added for managerial implications.

We agree that conducting additional interviews to gain additional insights into the potential collaborations between makerspaces and healthcare professionals is an interesting line of research. We have added this point as a suggestion for future research in the end of the article, as we believe such research could standalone as new article in the near future

 A small correction to the response above. The added references were:  Lemu (2019), Jung et al. (2023), Open Source Medical Supplies (n.d.), Osterwalder A & Pigneur Y. (2010),  and Pereira, T., Kennedy, J. V., & Potgieter, J. (2019).
